# Evaluation of the efficacy and safety of tegileridine at different doses in patients with postoperative pain: a meta-analysis of randomized controlled trials

**DOI:** 10.3389/fpain.2026.1860136

**Published:** 2026-07-03

**Authors:** Shanhui Yuan, Hui Ji, Yuntao Lu, Zhenyu Li, Junnan Wang, Hui Tang

**Affiliations:** 1 Stem Cell Clinical Research Center, Provincial Hospital Affiliated to Shandong First Medical University, Jinan, China; 2 Department of Pharmacy, Provincial Hospital Affiliated to Shandong First Medical University, Jinan, China; 3 Pain Management Department, Provincial Hospital Affiliated to Shandong First Medical University, Jinan, China

**Keywords:** acute pain, biased agonist, efficacy, safety, tegileridine

## Abstract

**Objective:**

This study aims to comprehensively evaluate the efficacy and safety of tegileridine at different doses in patients with postoperative acute pain through a meta-analysis.

**Methods:**

A systematic literature search was conducted across six databases from their inception to 24 March 2026 to identify all randomized controlled trials (RCTs) involving tegileridine for the treatment of postoperative acute pain. Meta-analyses were performed using RevMan 5.4 software.

**Results:**

A total of four cohorts from three RCTs involving 1,011 patients were included. Compared with placebo, tegileridine significantly reduced rSPID24 and rSPID12, demonstrating superior analgesic efficacy. Compared with morphine, there was no significant difference at rSPID12; however, tegileridine exhibited significantly weaker efficacy than morphine at rSPID24. Safety analyses revealed that although the total adverse events with tegileridine were higher than with placebo, it remained comparable to that of morphine. The incidence of nausea and vomiting with tegileridine was similar to that with placebo and significantly lower than that with morphine. No significant difference was observed in the incidence of respiratory depression between tegileridine and morphine.

**Conclusion:**

This meta-analysis demonstrates that the efficacy of tegileridine was significantly superior to that of placebo, and it had a more favorable safety profile than morphine regarding nausea and vomiting, suggesting its potential as a viable alternative for postoperative acute pain.

**Systematic Review Registration:**

[https://www.crd.york.ac.uk/prospero/view/CRD420261364062/1/0], PROSPERO CRD420261364062.

## 1 Introduction

Postoperative acute pain management remains a significant clinical challenge in perioperative medicine (1). Surveys indicate that approximately 75% of surgical patients experience moderate to severe acute pain (2), with nearly half receiving inadequate analgesia. Inadequate pain control following surgery adversely affects patients’ quality of life, increases healthcare costs, and enhances the risk of progression to chronic pain (3).

Opioids, the cornerstone for treating moderate to severe pain (4), produce analgesic effects primarily through the activation of μ-opioid receptors (MORs). However, their concurrent activation of the β-arrestin-2 signaling pathway induces adverse effects such as respiratory depression, nausea and vomiting, and gastrointestinal dysfunction, which substantially limits their clinical utility (5). Recently, the introduction of biased opioid receptor agonists has emerged as a promising strategy for pain management. These agents selectively activate G protein-coupled pathways to mediate analgesia, while minimizing β-arrestin-2 pathway activation, theoretically offering comparable analgesic efficacy with reduced adverse effects (6, 7).

Tegileridine (8) is a novel biased MOR agonist independently developed by Jiangsu Hengrui Pharmaceuticals. Approved in China in January 2024 for the treatment of moderate to severe acute pain, it exhibits high selectivity for MOR (EC_50_ = 4.3 nM) and activates the β-arrestin-2 pathway at approximately 10% of the level induced by morphine (9). Nevertheless, current clinical evidence on tegileridine remains limited, with no systematic and comprehensive evaluation. Therefore, this study aims to conduct a meta-analysis to integrate existing randomized controlled trial data, thereby providing a comprehensive assessment of the efficacy and safety of tegileridine in postoperative pain management and offering an evidence-based reference for clinical decision-making.

## 2 Methods

The systematic review and meta-analysis were conducted in accordance with the Preferred Reporting Items in Systematic Reviews and Meta-Analysis (PRISMA) guidelines (10). In addition, we conducted all the steps of this study according to the guidelines of the Cochrane System Review and Meta-analysis Manual. The methods were prespecified in a protocol with the International Prospective Register of Systematic Reviews (PROSPERO, CRD420261364062).

### 2.1 Literature search strategy

From database inception to March 2026, systematic searches were conducted in both English and Chinese databases, including PubMed, Scopus, the Cochrane Central Register of Controlled Trials (CENTRAL), Web of Science, CNKI, and Wanfang. Search terms included “SHR5884”, “Tegileridine”, “acute pain”, and “post-operative pain”. Boolean operators were used to combine subject headings and free-text terms for comprehensive retrieval.

### 2.2 Inclusion and exclusion criteria

All included randomized controlled trials (RCTs) met the following PICO criteria: Population: patients with postoperative pain; Interventions [loading dose (mg)/maintenance dose (mg)]: tegileridine treatment (1.0/0.05 mg/mg, 1.0/0.1 mg/mg, 1.0/0.2 mg/mg, 0.75/0.05 mg/mg); Comparators: placebo or morphine(3.0/1.0 mg/mg); Outcomes: Numeric Rating Scale (NRS) scores; the resting summed pain intensity difference over 24 or 12 h (rSPID24 or rSPID12); total incidence of adverse events; incidence of respiratory depression; incidence of nausea and vomiting. The exclusion criteria were as follows: duplicate publications, review articles, studies involving healthy volunteers, published study protocols without outcome data, and studies with outcomes unrelated to this analysis.

### 2.3 Literature screening and data extraction

Literature search and screening were performed independently by two researchers to ensure accuracy in study selection. Disagreements were resolved through discussion or by consultation with a third researcher. Extracted information included the first author, publication year, study period, sample size, intervention details, type of surgery, binding methods, and outcome measures.

This meta-analysis explored the efficacy of tegileridine in treating postoperative acute pain from both therapeutic and safety perspectives. The efficacy was mainly evaluated through rSPID24 and rSPID12. To calculate rSPID24 and rSPID12, the initial step involved determining the difference in pain intensity by subtracting the Numerical Pain Rating Scale (NPRS) score at each time point from the baseline. The safety analysis mainly involves comparing the proportion of patients reporting adverse events within 24 h after treatment between the tegileridine group and the placebo group, as well as the morphine group.

### 2.4 Quality assessment of included studies

The Cochrane Risk of Bias tool was applied to independently assess bias risk in each included study. The tool evaluates six domains of bias: randomization process, allocation concealment, blinding of participants and personnel, outcome assessment, incomplete outcome data, selective reporting, and other potential biases (11). Risk of bias in each domain was categorized as low, high, or unclear.

### 2.5 Statistical methods

Data analysis was conducted using RevMan 5.4 software. Continuous variables were analyzed using mean differences (MDs) with 95% confidence interval (CI), while dichotomous variables were analyzed using relative risks (RRs) with 95% CI. A value of *P* < 0.05 was considered statistically significant. RR values were calculated using the Mantel–Haenszel (M–H) method and MDs were calculated by the inverse variance method. Statistical heterogeneity was assessed using the chi-square test and *I*^2^ statistic. If *P* ≤ 0.10 and *I*^2^ ≥ 50%, substantial heterogeneity was deemed present, and a random-effects model was used for meta-analysis. Otherwise, a fixed-effects model was applied. Furthermore, the research results are interpreted through descriptive analysis. In this case, a subgroup analysis was conducted to compare the efficacy and safety of the commonly used doses of tegileridine, namely, 0.05, 0.1, and 0.2 mg.

## 3 Results

### 3.1 Literature search results

**Figure 1:**
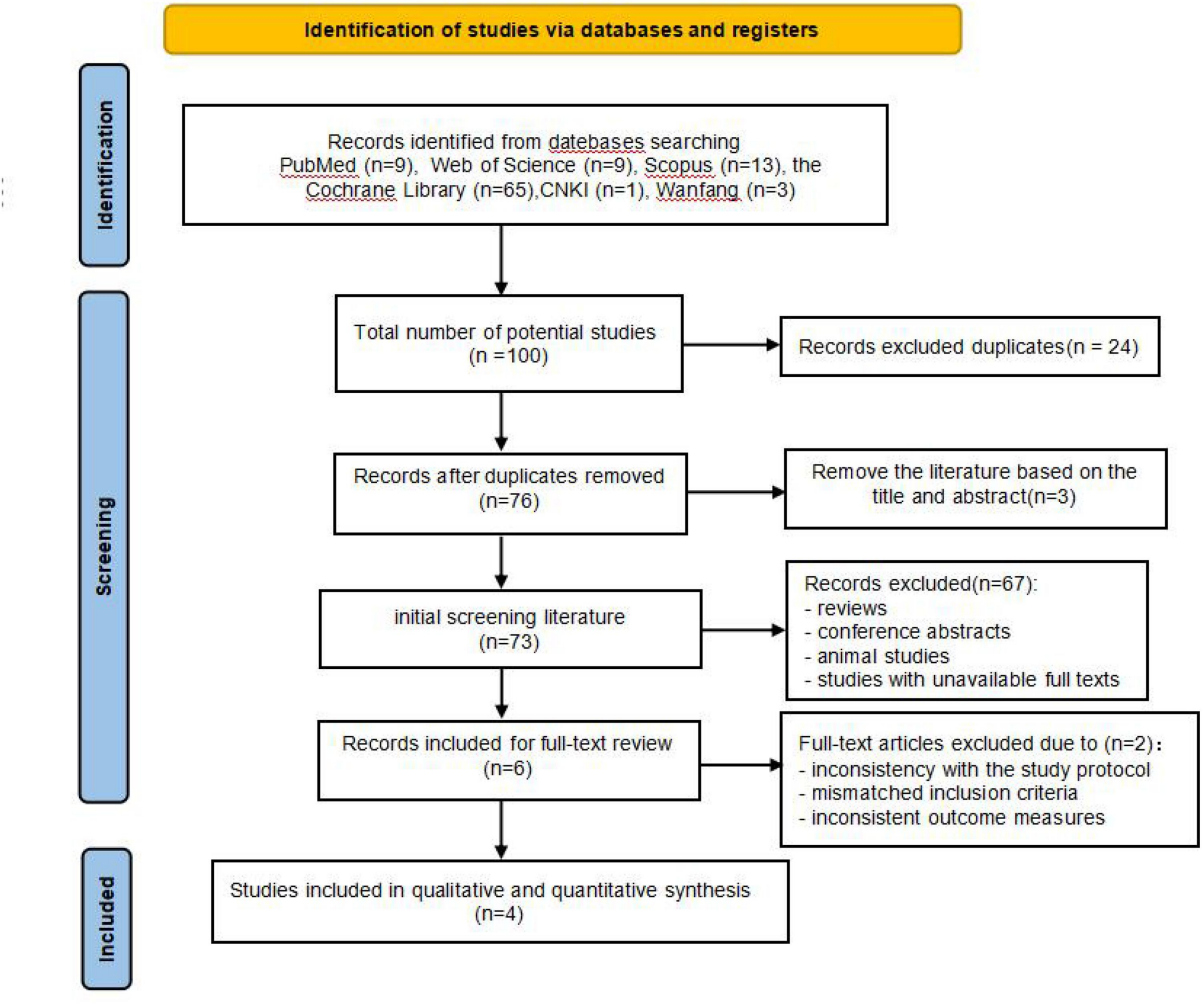
A PRISMA flow diagram.

After searching six medical databases, 100 articles were identified. After removing duplicates and screening titles and abstracts, 73 articles were assessed. Based on the inclusion and exclusion criteria, these 73 articles were rigorously evaluated. Ultimately, four cohorts from three RCTs (12–14) met the inclusion criteria for this meta-analysis, and the surgical types involved in our study were orthopedic surgery, abdominal surgery, and minimally invasive esophagectomy. The screening process is shown in Figure 1.

### 3.2 Quality assessment of included studies

**Figure 2:**
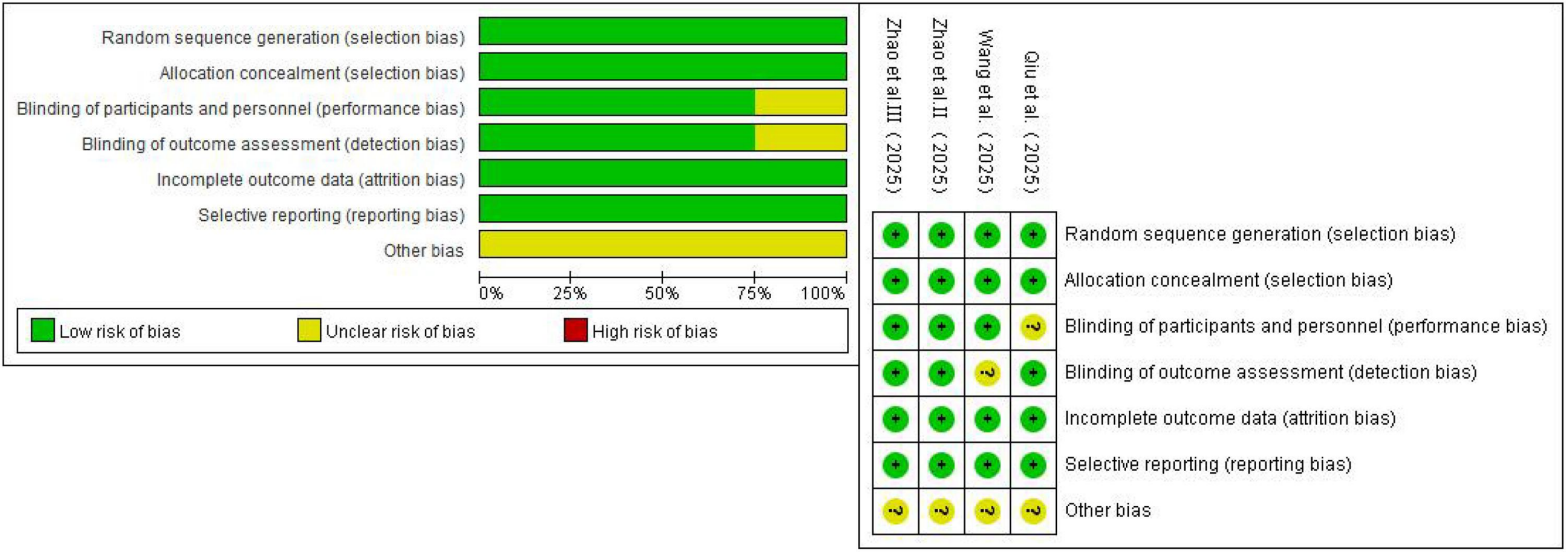
Risk of bias graph and summary.

According to the Cochrane Risk of Bias Assessment Tool, all included studies exhibited a low risk of selection bias. With regard to blinding of participants and personnel, one study did not report relevant details. In the evaluation of outcomes, three studies explicitly reported blinding of outcome assessors, while one study did not specify blinding of outcome evaluation. The risk of bias assessment for the included studies is summarized in Figure 2.

### 3.3 Basic characteristics of included studies

A total of four cohorts from three RCTs with postoperative pain were included, involving 1,011 patients. Specifically, 542, 211, and 258 patients were treated with tegileridine, placebo, and morphine, respectively. In these studies, each dose group of tegileridine was compared with placebo and morphine, respectively. The basic characteristics of the included studies are summarized in Table 1.

**Table 1:** Summary of the basic characteristics of included randomized controlled trials.

Study (year) identifier	Phase	Intervention (mg/mg)^a^ and sample size (*n*)	Female, *n* (%)	Age (years, mean ± SD)	BMI (kg/m^2^, mean ± SD)	Disease
Zhao et al. (2025) (12) CTR20220639	Ⅱ	Tegileridine 1.0/0.05 mg/mg (*n* = 30)	12 (40)	40.5 (18.2)	23.8 (2.9)	Orthopedic surgery
		Tegileridine 1.0/0.1 mg/mg (*n* = 31)	10 (32.3)	46.4 (19.6)	24.8 (2.5)	
		Tegileridine 1.0/0.2 mg/mg (*n* = 30)	14 (46.7)	46.4 (19.9)	24.1 (2.6)	
		Morphine 3.0/1.0 mg/mg (*n* = 30)	14 (46.7)	39.4 (20.0)	22.8 (2.4)	
Zhao et al. (2025) (12) CTR20220639	Ⅲ	Placebo (*n* = 80)	30 (37.5)	43.5 (17.4)	24.2 (2.6)	Orthopedic surgery
		Tegileridine 1.0/0.05 mg/mg (*n* = 79)	32 (40.5)	42.6 (18.2)	24.2 (2.4)	
		Tegileridine 1.0/0.1 mg/mg (*n* = 79)	36 (45.6)	43.9 (18.1)	23.9 (2.6)	
		Morphine 3.0/1.0 mg/mg (*n* = 81)	33 (40.7)	41.4 (18.3)	24.7 (2.2)	
Wang et al. (2025) (13) _	Ⅲ	Placebo (*n* = 131)	116 (88.5)	40.8 (10.5)	23.2 (2.5)	Abdominal surgery
		Tegileridine 0.75/0.05 mg/mg (*n* = 132)	121 (91.7)	42.7 (9.2)	23.1 (2.5)	
		Tegileridine 1.0/0.05 mg/mg (*n* = 131)	122 (93.1)	43.2 (9.4)	23.2 (2.4)	
		Morphine 3.0/1.0 mg/mg (*n* = 132)	126 (95.5)	41.2 (9.7)	23.2 (2.5)	
Qiu et al. (2025) (14) NCT06458400	—	Tegileridine 0.75/0.05 mg/mg (*n* = 30)	5 (16.7)	60 (8)	22.81 (2.98)	Minimally invasive esophagectomy

a mg/mg: loading dose/maintenance dose.

### 3.4 Comparison of tegileridine with placebo and morphine in terms of NRS scores

**Figure 3:**
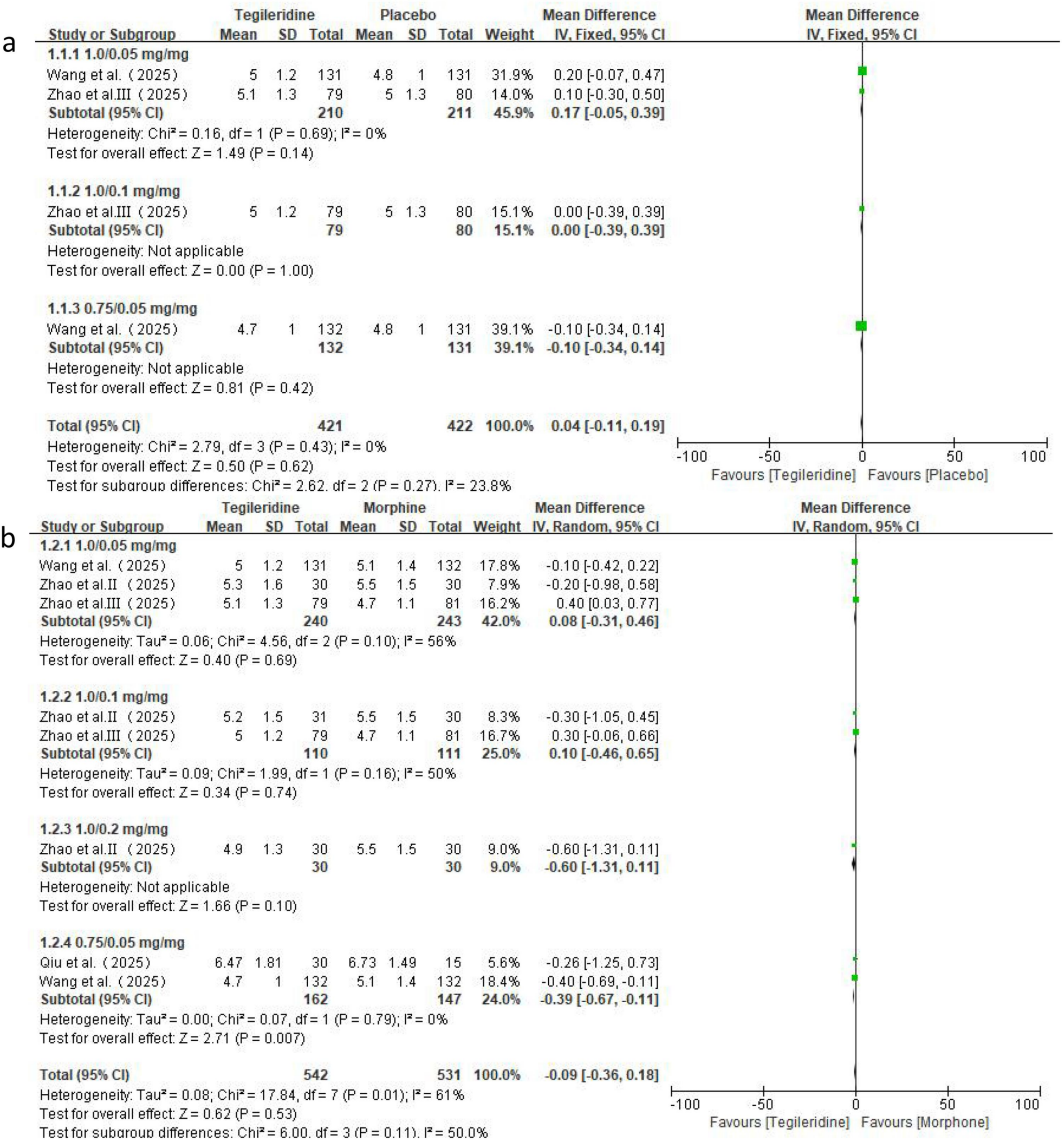
Changes in NRS scores of different doses of tegileridine. **(a)** Compared with placebo and **(b)** compared with morphine.

For the comparison of tegileridine vs. placebo, a fixed-effects model was used with subgroup analysis to explore heterogeneity. Two studies (12, 13) reported NRS score comparisons between tegileridine and placebo. The pooled study showed no heterogeneity (*I*^2^ = 0%, *P* = 0.43). The pooled analysis showed no significant difference in NRS scores between tegileridine and placebo (MD = 0.04, 95% CI −0.11 to 0.19, *P* = 0.62). A subgroup analysis by dose revealed no significant difference for tegileridine 1.0/0.05 mg/mg (MD = 0.17, 95% CI −0.05 to 0.39, *P* = 0.14), 1.0/0.1 mg/mg (MD = 0.00, 95% CI −0.39 to 0.39, *P* = 1.00), and 0.75/0.05 mg/mg (MD = −0.10, 95% CI −0.34 to 0.14, *P* = 0.42) compared with placebo, as shown in Figure 3.

For the comparison of tegileridine vs. morphine, a random-effects model was used with the subgroup analysis to explore heterogeneity. Four studies (12–14) reported NRS score comparisons between tegileridine and morphine. The pooled study showed substantial heterogeneity (*I*^2^ = 61%, *P* = 0.01). The subgroup analysis suggested that the 1.0/0.05 mg/mg and 1.0/0.1 mg/mg dose groups contributed to heterogeneity. The pooled analysis showed no significant difference in NRS scores between tegileridine and morphine (MD = −0.09, 95% CI −0.36 to 0.18, *P* = 0.53). The subgroup analysis by dose showed no significant difference for tegileridine 1.0/0.05 mg/mg (MD = 0.08, 95% CI −0.31 to 0.46, *P* = 0.69), 1.0/0.1 mg/mg (MD = 0.10, 95% CI −0.46 to 0.65, *P* = 1.00), and 1.0/0.2 mg/mg (MD = −0.60, 95% CI −1.31 to 0.11, *P* = 0.10) compared with morphine, while the 0.75/0.05 mg/mg dose group demonstrated significantly lower NRS scores than morphine (MD = −0.39, 95% CI −0.67 to −0.11, *P* = 0.007), as shown in Figure 3.

### 3.5 Comparison of tegileridine with placebo and morphine in terms of rSPID24

**Figure 4:**
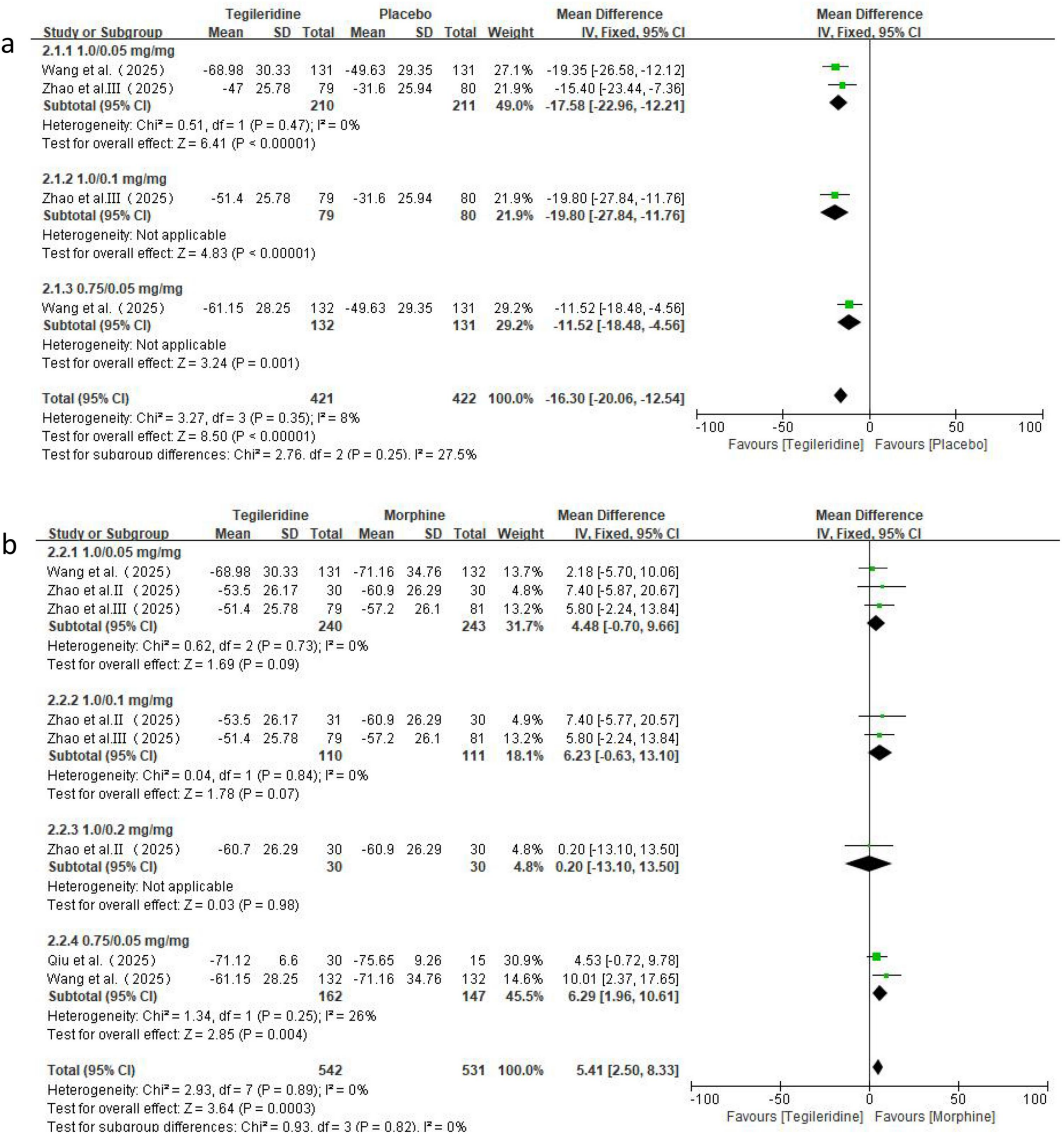
Changes in rSPID24 with different doses of tegileridine. **(a)** Compared with placebo and **(b)** compared with morphine.

For the comparison of tegileridine vs. placebo, a fixed-effects model was used with the subgroup analysis to explore heterogeneity. Two studies (12, 13) reported rSPID24 changes between tegileridine and placebo. The pooled study showed low heterogeneity (*I*^2^ = 8%, *P* = 0.35), indicating minimal heterogeneity. The pooled analysis showed a significant difference in rSPID24 between tegileridine and placebo (MD = −16.3, 95% CI −20.06 to −12.54, *P* < 0.00001). The subgroup analysis by dose revealed significant differences between tegileridine and placebo [1.0/0.05 mg/mg (MD = −17.58, 95% CI −22.96 to −12.21, *P* < 0.00001), 1.0/0.1 mg/mg (MD = −19.80, 95% CI −27.84 to −11.76, *P* < 0.00001), and 0.75/0.05 mg/mg (MD = −11.52, 95% CI −18.48 to −4.56, *P* = 0.001)], as shown in Figure 4.

For the comparison of tegileridine vs. morphine, a fixed-effects model was used with the subgroup analysis to explore heterogeneity. Four studies (12–14) reported rSPID24 changes between tegileridine and morphine. The pooled study showed no heterogeneity (*I*^2^ = 0%, *P* = 0.89). The pooled analysis showed a significant difference in rSPID24 changes between tegileridine and morphine (MD = 5.41, 95% CI 2.50–8.33, *P* = 0.0003), with tegileridine demonstrating inferior analgesic efficacy compared with morphine. The subgroup analysis based on dose revealed no statistically significant differences between tegileridine and morphine [1.0/0.05 mg/mg (MD = 4.48, 95% CI −0.70 to 9.66, *P* = 0.09), 1.0/0.1 mg/mg (MD = 6.23, 95% CI −0.63 to 13.10, *P* = 0.07), and 1.0/0.2 mg/mg (MD = 0.20, 95% CI −13.10 to 13.50, *P* = 0.98)], while the 0.75/0.05 mg/mg dose exhibited a significant difference (MD = 6.29, 95% CI 1.96–10.61, *P* = 0.004), with tegileridine demonstrating inferior analgesic efficacy compared with morphine, as shown in Figure 4.

### 3.6 Comparison of tegileridine with placebo and morphine in terms of rSPID12

**Figure 5:**
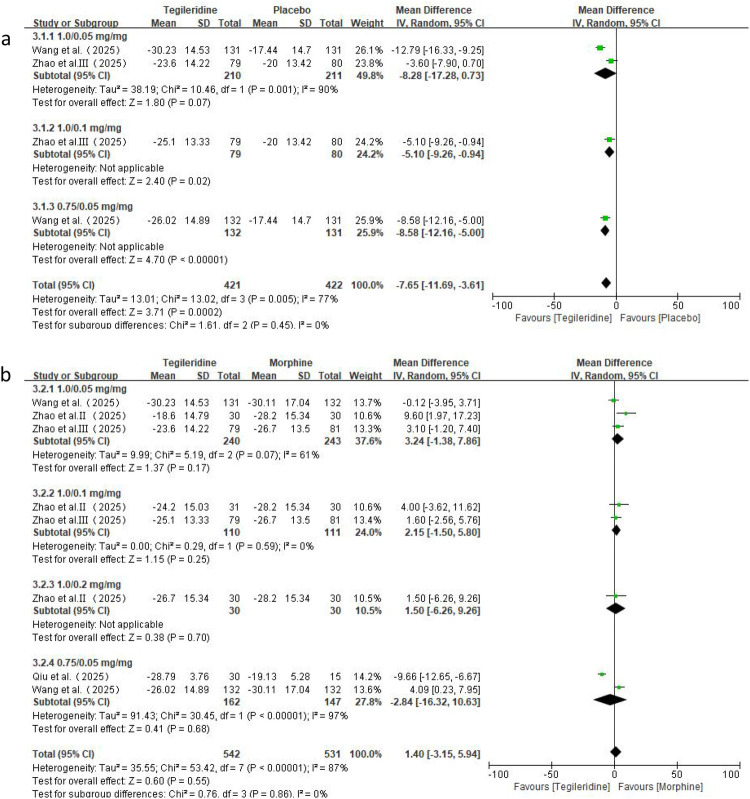
Changes in rSPID12 with different doses of tegileridine. **(a)** Compared with placebo and **(b)** compared with morphine.

Two studies (12, 13) reported the rSPID12 changes between tegileridine and placebo. The pooled study showed substantial heterogeneity (*I*^2^ = 77%, *P* = 0.005). The subgroup analysis identified the 1.0/0.05 mg/mg dose group as the main source of heterogeneity. The pooled analysis showed a significant difference in rSPID12 between tegileridine and placebo (MD = −7.65, 95% CI −11.69 to −3.61, *P* = 0.0002). The subgroup analysis based on dose revealed that there was no significant difference between tegileridine 1.0/0.05 mg/mg (MD = −8.28, 95% CI −17.28 to 0.73, *P* = 0.07) and placebo, while tegileridine doses of 1.0/0.1 mg/mg (MD = −5.10, 95% CI −9.26 to −0.94, *P* = 0.02) and 0.75/0.05 mg/mg (MD = −8.58, 95% CI −12.16 to −5.00, *P* < 0.00001) were significantly better than that of placebo (see Figure 5).

Four studies (12–14) reported the rSPID12 changes between tegileridine and morphine. The pooled study showed substantial heterogeneity (*I*^2^ = 87%, *P* < 0.00001). The subgroup analysis identified the 0.75/0.05 mg/mg dose group as the main source of heterogeneity, with notable differences in the proportion of female patients between studies. The pooled analysis showed no significant difference in rSPID12 changes between tegileridine and morphine (MD = 1.40, 95% CI −3.15 to 5.94, *P* = 0.55). The subgroup analysis based on dose revealed no significant difference between tegileridine and morphine [1.0/0.05 mg/mg (MD = 3.24, 95% CI −1.38 to 7.86, *P* = 0.17), 1.0/0.1 mg/mg (MD = 2.15, 95% CI −1.50 to 5.80, *P* = 0.25), 1.0/0.2 mg/mg (MD = 1.50, 95% CI −6.26 to 9.26, *P* = 0.70)] (see Figure 5).

### 3.7 Comparison of tegileridine with placebo and morphine in terms of total adverse events

**Figure 6:**
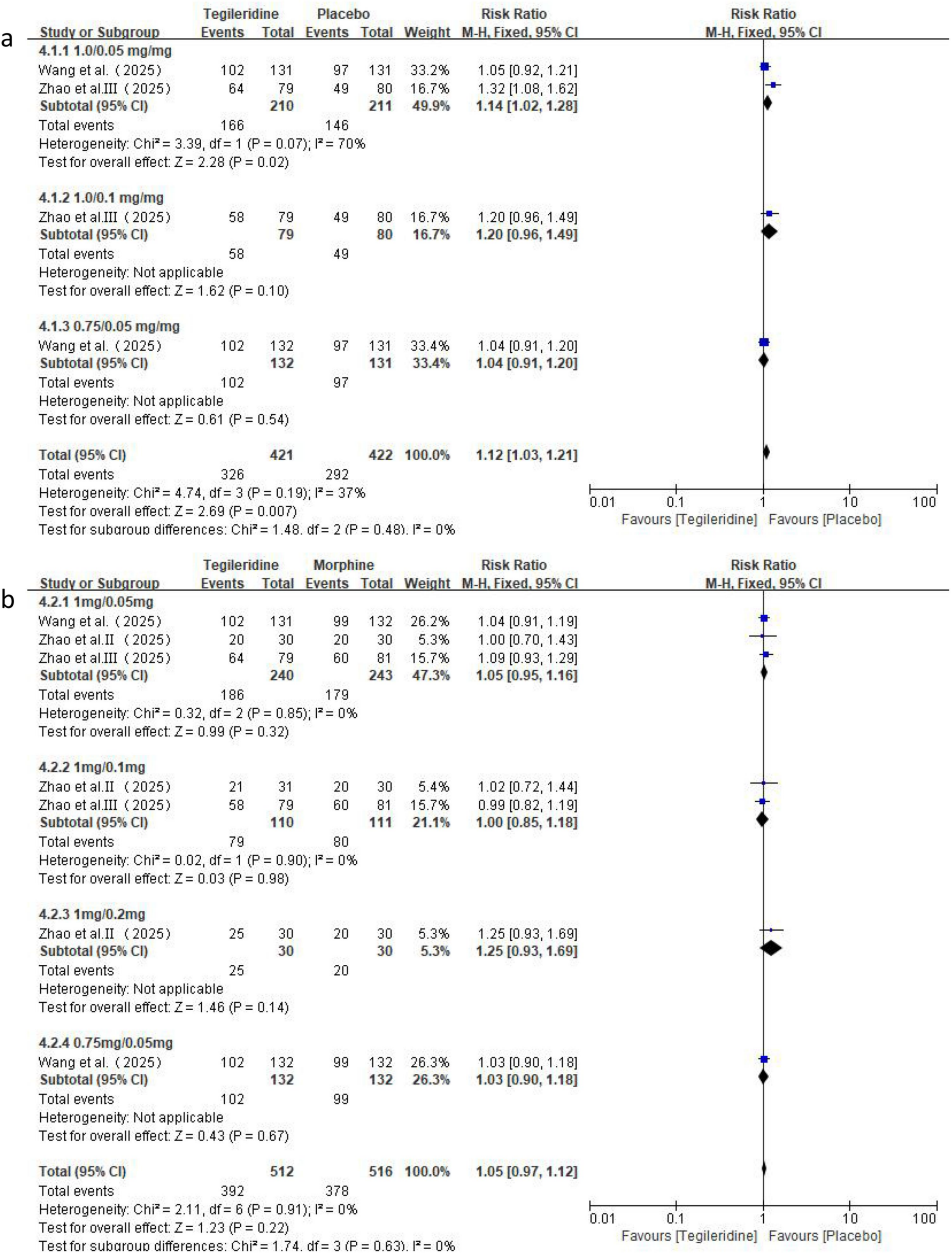
Comparison of total adverse reactions of different doses of tegileridine. **(a)** Compared with placebo and **(b)** compared with morphine.

Four studies (12–14) reported the total adverse events between tegileridine and placebo. The pooled study showed a certain degree of heterogeneity (*I*^2^ = 37%, *P* = 0.19). The pooled analysis showed a significant difference in total adverse events between tegileridine and placebo (RR = 1.12, 95% CI 1.03–1.21, *P* = 0.007), and the adverse events of tegileridine were higher than those of placebo. The subgroup analysis based on dose showed that there was a significant difference between tegileridine 1.0/0.05 mg/mg (RR = 1.14, 95% CI 1.02–1.28, *P* = 0.02) and placebo, while tegileridine 1.0/0.1 mg/mg (RR = 1.20, 95% CI 0.96–1.49, *P* = 0.10) and 0.75/0.05 mg/mg (RR = 1.04, 95% CI 0.91–1.20, *P* = 0.54) revealed no significant difference compared with placebo, as shown in Figure 6.

Four studies (12–14) reported the total adverse reactions between tegileridine and morphine. The pooled study showed no heterogeneity among the studies (*I*^2^ = 0%, *P* = 0.91). The pooled analysis showed no significant difference in total adverse events between tegileridine and morphine (RR = 1.05, 95% CI 0.97–1.12, *P* = 0.22). The subgroup analysis based on dose revealed that tegileridine of 1.0/0.05 mg/mg (RR = 1.05, 95% CI 0.95–1.16, *P* = 0.32), 1.0/0.1 mg/mg (RR = 1.00, 95% CI 0.85–1.18, *P* = 0.98), 1.0/0.2 mg/mg (RR = 1.25, 95% CI 0.93–1.69, *P* = 0.14), and 0.75/0.05 mg/mg (RR = 1.03, 95% CI 0.90–1.18, *P* = 0.67) did not significantly differ from that of morphine, as shown in Figure 6.

### 3.8 Comparison of tegileridine with placebo and morphine in terms of nausea and vomiting

**Figure 7:**
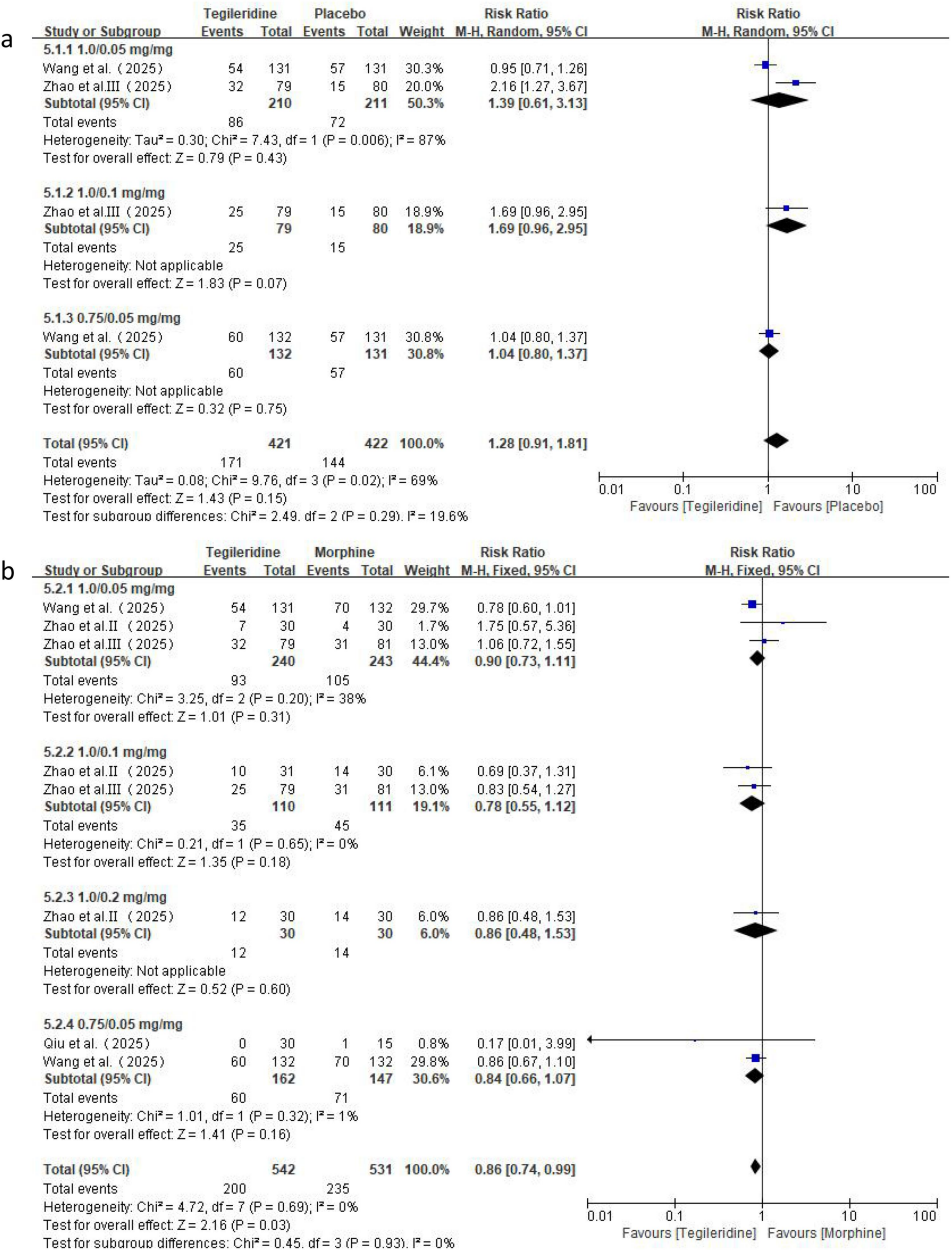
Comparison of the incidence of nausea and vomiting among different doses of tegileridine. **(a)** Compared with placebo and **(b)** compared with morphine.

Two studies (12, 13) reported the comparison of tegileridine with placebo for nausea and vomiting. The pooled study showed substantial heterogeneity (*I*^2^ = 69%, *P* = 0.02). The subgroup analysis by dose revealed that 1.0/0.05 mg/mg was the main source of heterogeneity. The pooled analysis showed no significant difference in the incidence of nausea and vomiting between tegileridine and placebo (RR = 1.28, 95% CI 0.91–1.81, *P* = 0.15). According to the dose, the subgroup analysis revealed that tegileridine of 1.0/0.05 mg/mg (RR = 1.39, 95% CI 0.61–3.13, *P* = 0.43), 1.0/0.1 mg/mg (RR = 1.69, 95% CI 0.96–2.95, *P* = 0.07), and 0.75/0.05 mg/mg (RR = 1.04, 95% CI 0.80–1.37, *P* = 0.75) did not significantly differ from that of placebo, as shown in Figure 7.

Four studies (12–14) reported the comparison of tegileridine with morphine for nausea and vomiting. The pooled study showed no heterogeneity (*I*^2^ = 0%, *P* = 0.69). The pooled analysis showed a significant difference in the incidence of nausea and vomiting between tegileridine and morphine (RR = 0.86, 95% CI 0.74–0.99, *P* = 0.03), with tegileridine having a lower incidence of nausea and vomiting than morphine. According to the dose, the subgroup analysis revealed that tegileridine 1.0/0.05 mg/mg (RR = 0.90, 95% CI 0.73–1.11, *P* = 0.31), 1.0/0.1 mg/mg (RR = 0.78, 95% CI 0.55–1.12, *P* = 0.18), 1.0/0.2 mg/mg (RR = 0.86, 95% CI 0.48–1.53, *P* = 0.60), and 0.75/0.05 mg/mg (RR = 0.84, 95% CI 0.66–1.07, *P* = 0.16) did not significantly differ from that of morphine, as shown in Figure 7.

### 3.9 Comparison of tegileridine with placebo and morphine in terms of respiratory depression

Only one study reported respiratory depression outcomes for the tegileridine vs. placebo comparison; therefore, this outcome was not pooled.

**Figure 8:**
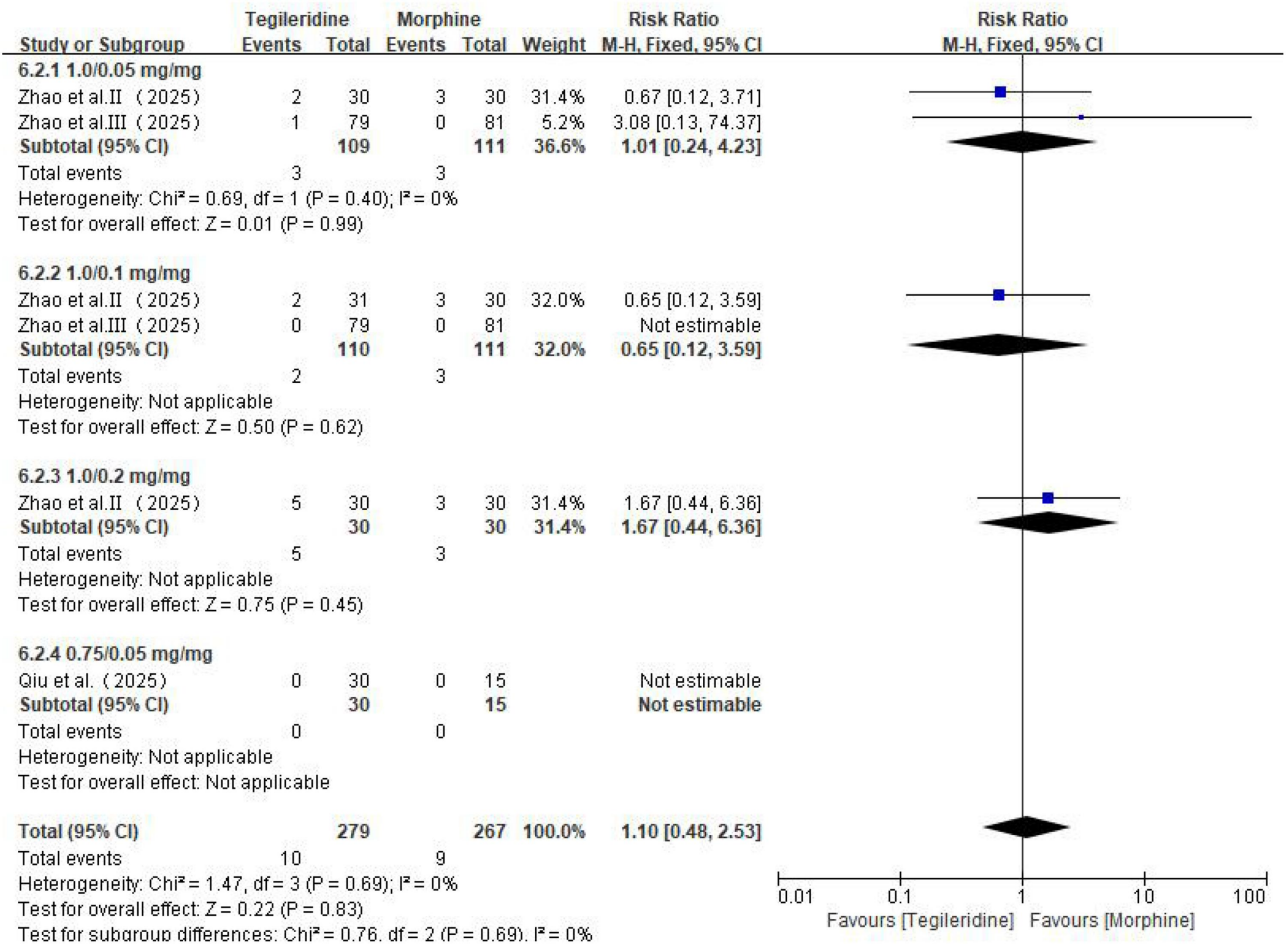
Comparison of the incidence of respiratory depression caused by different doses of tegileridine and morphine.

Three studies (12, 13) reported respiratory depression comparisons between tegileridine and morphine. The pooled study showed no heterogeneity among the studies (*I*^2^ = 0%, *P* = 0.69). The pooled analysis showed no significant difference in the incidence of respiratory depression between tegileridine and morphine (RR = 1.09, 95% CI 0.46–2.58, *P* = 0.85). The subgroup analysis showed that tegileridine doses of 1.0/0.05 mg/mg (RR = 0.94, 95% CI 0.21–4.26, *P* = 0.94), 1.0/0.1 mg/mg (RR = 0.65, 95% CI 0.12–3.59, *P* = 0.62), and 1.0/0.2 mg/mg (RR = 1.67, 95% CI 0.44–6.36, *P* = 0.45) were all not significantly different from that of morphine, while the 0.75/0.05 mg/mg dose could not be evaluated as no respiratory depression events occurred in either group, as shown in Figure 8.

### 3.10 Publication bias assessment

**Figure 9:**
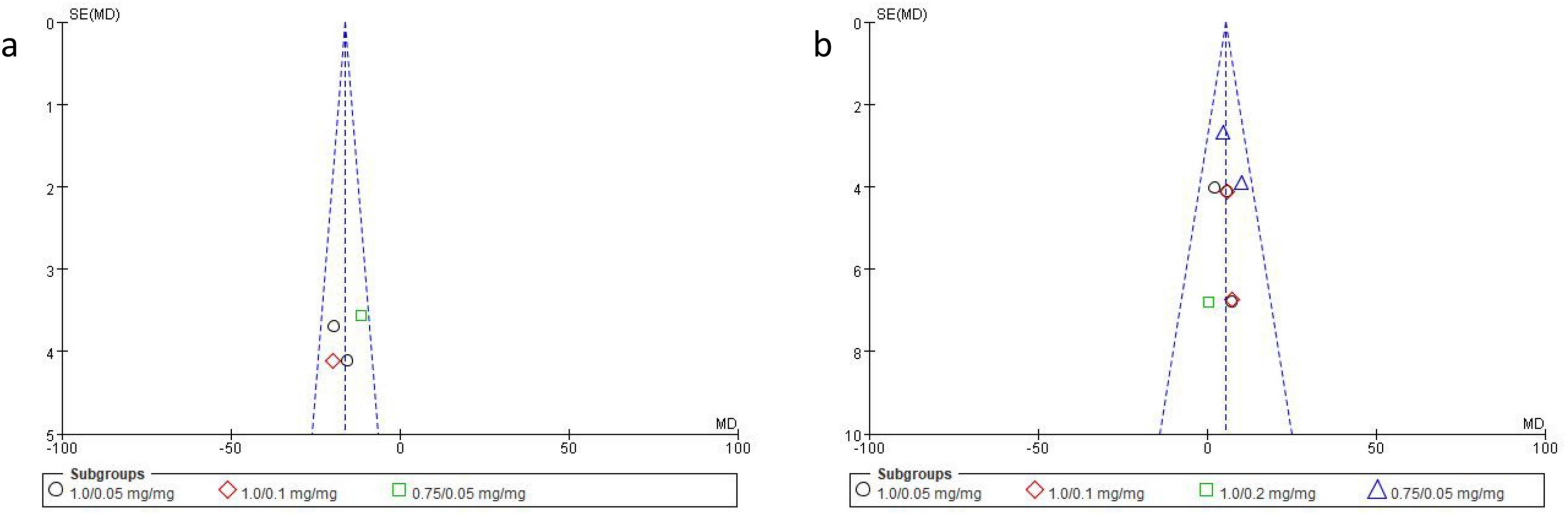
Funnel plot for assessing publication bias in the meta-analysis of rSPID24. **(a)** Versus placebo and **(b)** versus morphine.

To address the issue of publication bias, we assessed the bias using funnel plots for the primary outcomes of rSPID24. The funnel plot for rSPID24 between tegileridine and placebo (Figure 9a) did not indicate significant asymmetry, suggesting a low risk of publication bias. Similarly, the funnel plot for rSPID24 between tegileridine and morphine (Figure 9b) also did not show significant asymmetry. For other outcomes, including adverse effects, we performed similar assessments, and no significant publication bias was detected.

## 4 Discussion

In this systematic review and meta-analysis, we reviewed four cohorts from three independent studies evaluating tegileridine for postoperative acute pain (12–14). A total of 1,011 patients in the tegileridine treatment group, placebo treatment group, and morphine treatment group were included. Our aim was to examine the treatment effects and adverse events associated with the use of tegileridine vs. placebo or morphine in patients with postoperative acute pain.

According to our findings, tegileridine demonstrated comparable analgesic efficacy to morphine at 12 h (rSPID12: MD = 1.40, 95% CI −3.15 to 5.94, *P* = 0.55) but significantly weaker efficacy at 24 h (rSPID24: MD = 5.41, 95% CI 2.50 to 8.33, *P* = 0.0003). This time-dependent efficacy profile has important implications for clinical decision-making. The equivalent rSPID12 performance suggests that tegileridine may serve as an effective analgesic option for procedures with anticipated pain duration of less than 12 h or as a component of multimodal regimens where non-opioid adjuncts (e.g., Non-Steroidal Antiinflammatory Drugs (NSAIDs) or acetaminophen) are expected to provide sustained analgesia beyond the immediate postoperative period. However, for surgeries associated with prolonged moderate to severe pain (>24 h), the inferior rSPID24 performance indicates that tegileridine monotherapy may be insufficient, and either repeated dosing, Patient-Controlled Analgesia (PCA) dose adjustment, or a combination with longer-acting analgesics would be necessary. The observed 5.4-point difference in rSPID24, while statistically significant, reflects a measurable but modest reduction in cumulative analgesic effect over 24 h. Whether this magnitude of difference impacts patient-centered outcomes such as functional recovery, length of stay, or chronic pain development requires investigation in trials with longer follow-up and broader outcome measures.

Opioid-induced nausea and vomiting are among the most common adverse effects, and patients rank them as one of the most distressing postoperative complications. At all doses, the incidence of nausea and vomiting with tegileridine showed no significant difference compared with placebo. At the pooled dose level, tegileridine demonstrated a significantly lower incidence of nausea and vomiting than morphine (RR = 0.86, *P* < 0.05). However, at individual dose levels (0.05, 0.1, 0.2 mg), no significant differences were observed compared with morphine. This phenomenon may be attributed to insufficient sample sizes at individual dose levels, resulting in inadequate statistical power.

Opioid-induced respiratory depression (OIRD) is considered the most severe adverse event with potentially fatal consequences. As a novel biased opioid receptor agonist, the respiratory safety profile of tegileridine is of paramount importance. However, the current evidence remains insufficient to draw definitive conclusions regarding respiratory safety. Only one study reported respiratory depression outcomes for the tegileridine vs. placebo comparison, precluding pooled analysis. For the tegileridine vs. morphine comparison, while no statistically significant difference was observed (RR = 1.09, 95% CI 0.46–2.58, *P* = 0.85), the extremely low event rate (fewer than 10 events across all treatment arms) and wide confidence interval indicated that this analysis was substantially underpowered. Theoretically, tegileridine's minimal β-arrestin-2 activation (approximately 10% of morphine) suggests a potentially favorable central adverse effect profile, but this pharmacological premise has not been adequately translated into clinical evidence. Given the life-threatening nature of OIRD and the limited statistical power of the available data, clinicians should exercise caution and maintain standard respiratory monitoring protocols when using tegileridine, particularly in high-risk populations such as elderly patients, those with obstructive sleep apnea, or patients receiving concomitant central nervous system depressants.

This paper presents the first systematic review and meta-analysis of the efficacy and safety of tegileridine at different doses for postoperative acute pain, comparing tegileridine with placebo or morphine using high-quality meta-analysis methods. In addition, from a methodological perspective, we performed subgroup analyses to address heterogeneity wherever it existed in the outcome analysis.

Several limitations should be acknowledged when interpreting our findings. First, the evidence base remains relatively small, with only four studies involving 1,011 patients, and all studies originated from a single country (China). This may substantially affect the generalizability of our findings to broader surgical populations, particularly in regions with different ethnic backgrounds, healthcare systems, and perioperative management protocols. The lack of international multicenter data precludes definitive conclusions regarding the translatability of the efficacy and safety profiles of tegileridine to global clinical practice. Second, variations in surgical types (orthopedic, abdominal, and minimally invasive esophagectomy) and baseline patient characteristics across studies may have introduced confounding factors. Third, the follow-up period was limited to 24 h, precluding assessment of long-term efficacy and safety. Fourth, only one study addressed respiratory depression adverse events, resulting in limited outcome indicators and insufficient statistical power. Furthermore, despite subgroup analyses by dose, some outcome measures still exhibited substantial heterogeneity, which may reflect unmeasured differences in surgical protocols, anesthesia regimens, or patient populations across centers. Future studies should prioritize large-scale, international, multicenter phase III RCTs with extended follow-up periods and diverse surgical populations to validate our findings and enhance their external validity.

## 5 Conclusion

This systematic review and meta-analysis showed that in patients with postoperative pain, tegileridine treatment was superior to that of placebo in terms of analgesic efficacy at 12 and 24 h and comparable to that of morphine at 12 h. Although tegileridine showed slightly weaker efficacy than morphine at 24 h, it demonstrated a more favorable safety profile, particularly in terms of nausea and vomiting incidence. Additional phase III clinical trials with longer follow-up periods, diverse surgical populations, and special patient groups (such as elderly patients and those with hepatic or renal impairment) are needed to consolidate the efficacy and safety of tegileridine in the treatment of moderate to severe postoperative pain.

## Data Availability

The original contributions presented in the study are included in the article/Supplementary Material, and further inquiries can be directed to the corresponding author.
